# Primary vs. second primary cT1-T2 oral squamous cell carcinoma: comparing the outcomes

**DOI:** 10.3389/fsurg.2025.1610776

**Published:** 2025-07-24

**Authors:** Noëmi Ramirez-Guanche, Fien Jaeken, Davide Di Santo, Sandra Nuyts, Paul M. Clement, Annousschka Laenen, Jeroen Meulemans, Vincent Vander Poorten

**Affiliations:** ^1^ Department of Otorhinolaryngology-Head and Neck Surgery, University Hospitals Leuven, Leuven, Belgium; ^2^ Department of Oncology, Section of Head and Neck Oncology, Catholic University Leuven, Leuven, Belgium; ^3^ Department of Radiotherapy–Oncology, University Hospitals Leuven, Leuven, Belgium; ^4^ Department of Oncology, Laboratory Experimental Radiotherapy, Catholic University Leuven, Leuven, Belgium; ^5^ Department of Biostatistics and Statistical Bioinformatics Center, KU Leuven, Leuven, Belgium

**Keywords:** oral squamous cell carcinoma, oral cancer, head and neck cancer, second primary tumor, prognostic factors, cT1-T2

## Abstract

**Introduction and aim:**

Head and neck cancer (HNC) is the third most common cancer worldwide, with oral squamous cell carcinoma (OSCC) having the highest incidence. Despite early diagnosis in 50% of cases, recurrence and poor survival remain concerns. This study compares survival outcomes between primary and second primary cT1-T2 OSCC.

**Materials and methods:**

A single-center historical cohort study included 60 patients treated for cT1-T2 OSCC between 2010 and 2022. Patient demographics, tumor characteristics, and treatment modalities were collected. Treatment followed ESMO guidelines, primarily involving surgery with or without postoperative radiotherapy. Kaplan–Meier analysis and Cox proportional hazards models assessed overall survival (OS), disease-specific survival (DSS), and disease-free survival (DFS).

**Results:**

The 2- and 5-year OS rates were 85% and 64.9%, while DSS rates were 91.4% and 87.3%, respectively. Median OS was 7.4 years. Patients with primary tumors had significantly better OS (HR = 0.409, *p* = 0.038) and DFS (HR = 0.399, *p* = 0.036) than those with second primary tumors. Female patients had a 74.7% lower risk of death, and males had significantly shorter DFS (*p* = 0.024). Advancing tumor stage increased disease-specific mortality risk (HR = 1.737, *p* = 0.043). Multiple lymph node involvement correlated with worse OS (HR = 2.884, *p* = 0.031) and DFS (HR = 3.971, *p* = 0.006). Gross extranodal extension (ENE) was significantly associated with poorer OS (*p* = 0.048) and showed a borderline association with DFS (*p* = 0.050).

**Conclusion:**

This study confirms second primary malignancies as a key prognostic factor for survival in OSCC. Male sex, advanced TNM stage, gross ENE, multiple lymph node involvement, and active smoking status were linked to poorer outcomes. Larger studies with multivariate analysis comparing primary and non-primary tumors are needed to validate these findings.

## 1 Introduction

Head and neck cancer (HNC) ranks as the sixth most prevalent cancer worldwide, with approximately 900 000 new diagnosis and 450 000 deaths yearly (1). This accounted for roughly 4.7% of all cancers and 4.5% of all cancer-related deaths in 2020. In 2019, within HNC, cancer in the subsite “oral cavity” had the highest incidence with 373 098 (25.5% of the total number) patients diagnosed, leading to 199 398 deaths (1–3). In Europe, the main risk factors for its development are tobacco and alcohol consumption, and these have a multiplicative effect (4, 5). Although oral cavity squamous cell carcinoma (OCSCC) has historically shown a male predominance, the male-to-female ratio is gradually declining (2, 6). The 5-year survival rate for OCSCC is approximately 60% (4, 6).

Staging follows the International Union for Cancer Control (UICC) tumor-node-metastasis (TNM) classification, which facilitates a prognostic stratification and guides treatment selection (5). In the most recent 8th edition, the T-classification definition relies on the diameter of the tumor, but also on its depth of invasion (DOI), given the strong association of the latter with disease-specific survival (DSS). Similarly, extranodal extension (ENE) is an additional factor to the N-classification because of its negative prognostic impact (7). While approximately 50% of OCSCC are diagnosed at an early stage, allowing for timely surgical intervention, a subset of early-stage patients experiences recurrence and poor survival despite clear surgical margins and the absence of lymph node metastases (8, 9). In addition to the TNM classification at presentation, several patient- (age, sex, smoking and drinking habits), tumor- (tumor site, perineural and lymphovascular invasion, cell differentiation) and treatment-related factors (surgical margins, pathological findings in the neck dissection, postoperative radiotherapy) are associated with oncological outcomes (10).

The primary objective of this single-center, single-department historical cohort study was to verify our clinical impression that primarily occurring OCSCC is associated with better outcomes than OCSCC occurring as a second primary tumor (SPT) in patients previously treated for head and neck squamous cell carcinoma (HNSCC). At the same time, this study aimed at identifying other prognostic factors that are associated with a poor oncological outcome.

## 2 Materials and methods

### 2.1 Patients

Approval of this study was obtained from the Research Ethics Committee UZ/KU Leuven and OBC (MP023740). According to our institutional policy, informed consent concerning the retrospective use of patient information was not requested.

Between January 1, 2010, and December 31, 2022, 264 consecutive patients were treated for an OCSCC in the Department of Otorhinolaryngology-Head and Neck Surgery (ORLHNS). Of these, 40.4% had a cT1 or a cT2 (any N) lesion at presentation, after restaging according to the UICC 8th edition. Patients with tumors of the lip were excluded. Both patients with a first or a second primary OCSCC, treated with surgery and/or radiotherapy with curative intent, were included in the historical cohort for this study (total *n* = 60). Following a multidisciplinary team discussion, patients were treated according to the ESMO Guidelines (11). The primary treatment consisted of surgery with or without tailored postoperative radiotherapy, a minority of patients with a surgical contraindication underwent primary radiotherapy. Surgical treatment involved resection of the tumor with wide margins, with or without neck dissection, depending on tumor location, depth of invasion, and cN-status. Adjuvant treatment was administered depending on adverse pathological features, such as advanced pT-classification, positive or close margins, perineural, vascular, or lymphatic invasion, extensive nodal involvement (pN2 or pN3), or extranodal extension (11).

### 2.2 Methods

A historical cohort study was conducted. Data concerning patient-, tumor- and treatment characteristics were collected in a REDCap (Vanderbilt University, Nashville, Tennessee, US) electronic database (12, 13). These data then underwent initial coding in InfoPath (Microsoft Corp Redmond, Washington, US) followed by pseudo-anonymization, and storage in an online repository facilitated by REDCap.

### 2.3 Statistical analysis

Statistical analyses were performed using SAS software (version 9.4 of the SAS System for Windows). Descriptive statistics (mean, median, range, proportions) were calculated and a survival analysis (univariate: Kaplan–Meier—Log Rank testing) was performed for the oncological outcomes overall survival (OS), disease specific survival (DSS), disease free survival (DFS), and recurrence free survival (RFS). Variables and their definition used in the analysis are listed in Table 1.

**Table 1:** Variables in the analysis.

Variable	Coding and levels
Age	Linearly^a^ and categorized (<50, 50–59, 60–69, 70–79, ≥80)
Sex	Male vs. female
Pre-and post- treatment smoking status	Categorized [non-smoker, past smoker (>12 months), active smoker]
Pre- and post-treatment alcohol status	Categorized (never, occasional (men <14units/week; women <7units/week), active heavy drinker (men >14units/week; women >7units/week), past heavy drinker (>12 months)
Oral cavity subsite^d^	Categorized (tongue, floor of mouth, hard palate, buccal)
Primary tumor	Dichotomy: primary vs. second primary
cT classification (UICC, 8th edition)^6^	cT1 or cT2
cN classification (UICC, 8th edition)^6^	Linearly (cN0, cN1, cN2a, cN2b)
pT classification (UICC, 8th edition)^6^	pT1 or pT2
pN classification (UICC, 8th edition)^6^	Linearly (pN0, pN1, pN2a, pN2b, pN2c, pN3)
Stage (UICC, 8th edition)^6^	Linearly (I, II, III, Iva, IVb)
Number of positive lymph nodes^c^	Categorized (0, 1, > 1)
Depth of invasion^b^	Linearly
Degree of histological differentiation^b^	Well-, moderately- or poorly differentiated as described in the pathology report
pENE^c^	Categorized (no, minimal ≤2 mm, gross >2 mm)^14^
Perineural growth^b^	Dichotomy: described vs. not described in the pathology report
Lymphovascular invasion^b^	Dichotomy: described vs. not described in the pathology report
Surgical margins^b^	Categorized (negative >5 mm, close 1–5 mm, positive)

^a^ Linear analysis means we assumed the variable to be continuous, and we tested, e.g., for age whether the risk for recurrence increases linearly with every year the patient age; at the same time, we tested whether a categorized form of the variable fits better to the observed increase in risk with increasing age.

^b^ Only evaluated for patients who had surgery.

^c^ Only evaluated for patients who had a neck dissection.

^d^ Only subsites present in our material are mentioned.

## 3 Results

### 3.1 Descriptive analysis

There were 21 women (35%) and 39 men (65%) with a median age of 61.4 years (range, 14–89) at diagnosis, 68% presented with tongue cancer followed by 20% with floor of mouth cancer. Of these, 77% (*n* = 46) presented with a primary OCSCC and 23% (*n* = 14) of them presented with a second primary OCSCC after a previous treatment for HNSCC. SPT were defined using the criteria of Warren and Gates, as refined by Hong et al., requiring histological malignancy, separation by normal tissue, exclusion of metastasis from the index tumor, and—when histology is identical—either a ≥3-year interval or ≥2 cm of normal mucosa between tumors (14–16).

Patient-, tumor- and treatment characteristics are listed in Table 2. The clinical (c) and pathological (p) TNM classifications are presented in Table 3, as well as the stage grouping.

**Table 2:** Patient (A), tumor (B), and treatment (C) characteristics.

A		
Variable	Statistic	All
Age	N	60
	Mean	61.43
	Std	16.982
	Median IQR Range	62.50 (52.00; 75.00) (14.00; 89.00)
Sex		
Female	n/N (%)	21/60 (35.00%)
Male	n/N (%)	39/60 (65.00%)
Pre-treatment smoking status		
Non-smoker	n/N (%)	16/57 (28.07%)
Past smoker	n/N (%)	16/57 (28.07%)
Active smoker	n/N (%)	25/57 (43.86%)
Pre-treatment alcohol status		
Never	n/N (%)	6/54 (11.11%)
Occasional	n/N (%)	25/54 (46.30%)
Active heavy drinker	n/N (%)	19/54 (35.19%)
Past heavy drinker	n/N (%)	4/54 (7.41%)
Post-treatment smoking status		
Non-smoker	n/N (%)	16/56 (28.57%)
Past smoker	n/N (%)	28/56 (50.00%)
Active smoker	n/N (%)	12/56 (21.43%)
Post-treatment alcohol status		
Never	n/N (%)	6/54 (11.11%)
Occasional	n/N (%)	30/54 (55.56%)
Active heavy drinker	n/N (%)	10/54 (18.52%)
Past heavy drinker	n/N (%)	8/54 (14.81%)

B		
Primary vs. second primary		
Primary	n/N (%)	46/60 (76.67%)
Second primary	n/N (%)	14/60 (23.33%)
Oral subsite		
Tongue	n/N (%)	41/60 (68.33%)
Floor of mouth	n/N (%)	12/60 (20.00%)
Hard palate	n/N (%)	1/60 (1.67%)
Buccal mucosa	n/N (%)	6/60 (10.00%)
Histological differentiation		
Well	n/N (%)	5/57 (8.77%)
Moderately	n/N (%)	43/57 (75.44%)
Poor	n/N (%)	9/57 (15.79%)
Depth of invasion (DOI)	N	52
	Mean	7.14
	Std	5.820
	Median	5.50
	IQR	(3.00; 10.25)
	Range	(0.20; 25.00)
ENE		
No	n/N (%)	32/36 (88.89%)
Minimal	n/N (%)	3/36 (8.33%)
Gross	n/N (%)	1/36 (2.78%)
Perineural invasion		
No	n/N (%)	43/59 (72.88%)
Yes	n/N (%)	16/59 (27.12%)
Lymphovascular invasion		
No	n/N (%)	51/59 (86.44%)
Yes	n/N (%)	8/59 (13.56%)
Surgical margins		
negative	n/N (%)	13/59 (22.03%)
close	n/N (%)	42/59 (71.19%)
positive	n/N (%)	4/59 (6.78%)
C		
Primary treatment modality		
Surgery	n/N (%)	59/60 (98.33%)
Radiotherapy	n/N (%)	1/60 (1.67%)
Neck dissection		
None	n/N (%)	22/54 (40.47%)
Ipsilateral	n/N (%)	27/54 (50.00%)
Bilateral	n/N (%)	5/54 (9.26%)
Adjuvant treatment		
Radiotherapy	n/N (%)	13/60 (21.67%)
Chemoradiotherapy	n/N (%)	7/60 (11.67%)

**Table 3:** cTNM distribution (A), pTNM distribution (B) and stage.

A						
cTNM	cN0 (82%)		cN1 (7%)		cN2a (5%)	cN2b (7%)
cT1						
*n* *=* *29 (48%)*	25		1		1	2
cT2						
*n* *=* *31 (52%)*	24		3		2	2
B						
pTNM	pN0 (78%)	pN1 (3%)	pN2a (3%)	pN2b (7%)	pN2c (3%)	pN3 (3%)
pT1						
*n* *=* *28 (47%)*	25	0	0	1	0	2
pT2						
*n* *=* *26 (43%)*	21	0	2	3	0	0
pT3						
*n* *=* *6 (10%)*	1	2	0	1	2	0
				*Stage I*		
				*Stage II*		
				*Stage III*		
				*Stage IVa*		
				*Stage IVb*		

### 3.2 Overall survival and recurrence

**Figure 1:**
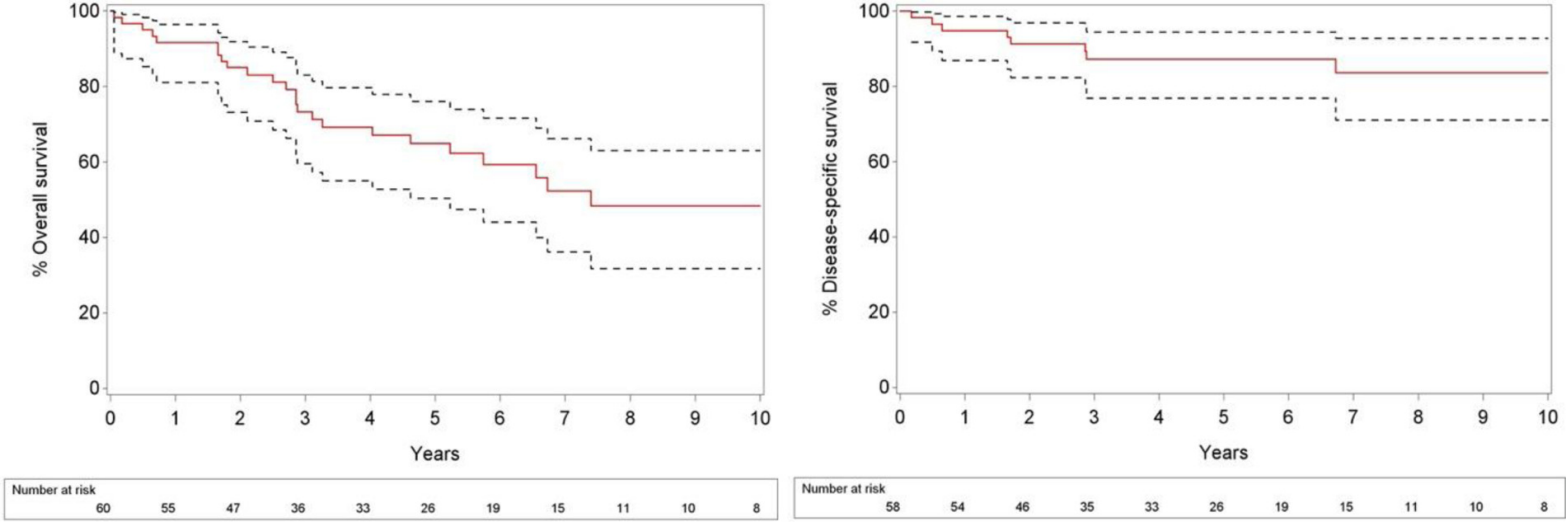
The 10-year cumulative OS (left) and DSS (right) of 60 patients with OCSCC.

At the time of analysis, 26 patients (43.3%) had died. Of these patients, 8 (13.3%) died of their disease, 16 (26.7%) from another cause and 2 of an unknown cause. 34 patients were continuing follow-up. The median follow-up was 5.56 years (range, 0.06–13.25), and follow-up for patients alive at end of follow-up was 5.28 years (range, 1.81–13.25). Overall 2- and 5 year survival rates for all 60 patients from the day of diagnosis were 85% and 64.9%, respectively, and 2- and 5-year rates of disease specific survival from the day of diagnosis were 91.4% and 87.3%, respectively (Figure 1). The median overall survival was 7.4 years.

Recurrence occurred in 22 patients (37.9%), including 18 with locoregional recurrence and 4 with distant metastases. The 2- and 5-year recurrence-free survival rates for all 60 patients from the time of diagnosis were 80% and 62%, respectively. During follow-up after treatment for the initial malignancy, eight patients (13.3%) developed a new primary tumor: three patients developed a SPT, and five patients who initially presented with a SPT developed a third primary tumor.

Among the 14 patients who presented with a SPT, 50% (*n* = 7) remained recurrence-free during follow-up, while two patients (14.3%) developed a recurrence—one local recurrence after 6 months and one regional recurrence in the neck after 16 months. Additionally, 5 patients (35.7%) developed a third primary tumor at a completely different anatomical subsite, which occurred at 5, 6, 12, 34, and 90 months following treatment for the SPT (median: 12 months).

### 3.3 Prognostic factors for outcomes

In this cohort, females have a 74.7% lower risk of death compared to males [HR 0.25 (95% CI: 0.08–0.86), *p* = 0.027] and DFS is significantly worse in males (*p* = 0.024). Active smokers have a higher risk of death, compared to past smokers and non-smokers (*p* = 0.036).

**Figure 2:**
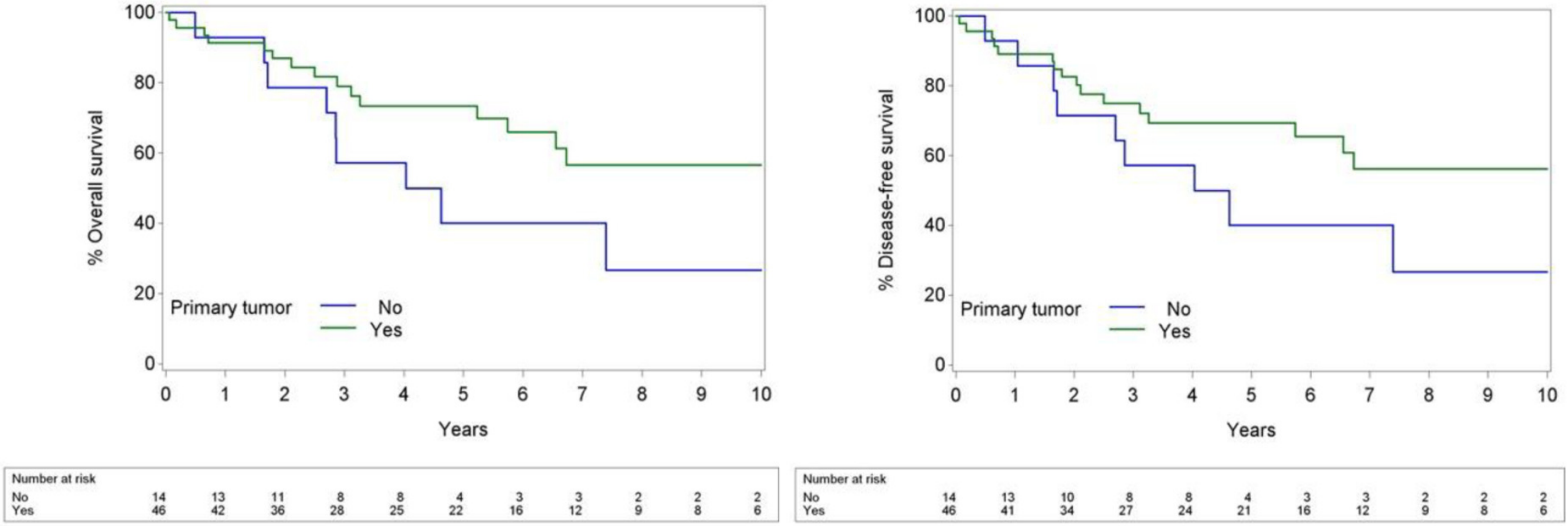
Kaplan–Meier curves of primary compared to second primary OCSCC for OS (left) and DFS (right).

Patients with OCSCC as a primary tumor had significantly better OS compared to those with OCSCC presenting as a SPT, with a HR of 0.41 (*p* = 0.038). This indicates a 59.1% reduction in the risk of death for patients with OCSCC as a primary tumor. Furthermore, patients with primary OCSCC had significantly better DFS compared to those with second primary OCSCC, with a HR of 0.40 [(95% CI: 0.17–0.94), *p* = 0.036]. Kaplan–Meier curves for OS and DFS are shown in Figure 2.

An increase in tumor stage by one level was associated with a significantly higher risk of disease-specific mortality, with a hazard ratio of 1.74 [(95% CI: 1.02–2.96), *p* = 0.043], indicating that each level up in stage is linked to a 73.7% higher risk of death due to disease.

Compared to no lymph node involvement, having one affected lymph node does not significantly impact OS, DSS, of DFS. However, involvement of more than one lymph node is significantly associated with worse OS (HR = 2.88, *p* = 0.031) and DFS (HR = 3.97, *p* = 0.006), while no significant association is observed for DSS (*p* = 0.120).

Compared to no ENE, gross ENE was significantly associated with worse OS [HR = 10.13, (95% CI: 1.02–100.36), *p* = 0.048] and showed a borderline association with worse DFS [HR = 11.02, (95% CI: 0.10–121.86), *p* = 0.050]. Minimal ENE was not significantly associated with OS or DSS but was strongly associated with worse DFS [HR = 59.31, (95% CI: 4.72–745.08), *p* = 0.002] (Table 4).

**Table 4:** Survival analysis of the oral squamous cell carcinoma patients.

Variables	Test	OS		DSS		DFS	
		*HR (95% CI)*	*p-value*	*HR (95% CI)*	*p-value*	*HR (95% CI)*	*p-value*
Age	+1 year	1.02 (1.00;1.05)	0.101	1.01 (0.96;1.05)	0.855	1.02 (1.00;1.05)	0.188
Sex	Female vs male	0.25 (0.08;0.86)	**0**.**027**	0.32 (0.04;2.76)	0.299	0.25 (0.07;0.83)	**0**.**024**
Smoking status *(Ref = non-smoker)*	Global		0.111		0.640		0.153
	Active	5.44 (1.12;26.52)	**0**.**036**	1.62 (0.17;15.68)	0.676	4.28 (0.97;18.85)	0.055
	Past	3.43 (0.77;15.20)	0.105	3.03 (0.27;34.02)	0.369	4.07 (0.78;21.15)	0.095
Subsite *(Ref = Tongue)*	Floor of mouth	1.41 (0.54;3.69)	0.488	1.00 (0.10;9.68)	0.999	1.88 (0.74;4.82)	0.187
	Buccal mucosa	1.33 (0.38;4.70)	0.660	4.30 (0.71;26.01)	0.112	1.60 (0.44;5.78)	0.474
Primary tumor	Yes vs. no	0.41 (0.18;0.95)	**0**.**038**	0.54 (0.10;2.97)	0.478	0.40 (0.17;0.94)	**0**.**036**
T status	T2 vs. T1	1.31 (0.56;3.10)	0.536	0.95 (0.16;5.67)	0.952	1.29 (0.53;3.19)	0.576
N status	>N0 vs. N0	1.64 (0.55;4.93)	0.377	1.30 (0.15;11.27)	0.813	2.01 (0.66;6.11)	0.221
UICC Stage grouping	+1	1.18 (0.84;1.66)	0.336	1.74 (1.02;2.96)	**0**.**043**	1.28 (0.91;1.81)	0.155
Lymph node burden *(Ref = 0)*	1	0.46 (0.06;3.51)	0.457	2.25 (0.23;21.64)	0.483	0.54 (0.07;4.09)	0.548
	>1	2.88 (1.10;7.53)	**0**.**031**	4.17 (0.69;25.27)	0.120	3.97 (1.50;10.51)	**0**.**006**
Lowest level positive lymph node	+1	0.66 (0.34;1.29)	0.224	0.98 (0.34;2.78)	0.963	0.42 (0.16;1.09)	0.075
Histological differentiation *(Ref = Well)*	Moderate	0.94 (0.12;7.29)	0.952	0.29 (0.03;2.86)	0.289	1.07 (0.14;8.38)	0.946
	Poor	1.00 (0.10;9.80)	0.998	0.35 (0.02;5.98)	0.471	1.68 (0.19;15.23)	0.644
ENE *(Ref = no ENE)*	Gross	10.13 (1.02;100.4)	**0**.**048**	–	0.997	11.02 (1.00;121.86)	0.050
	Minimal	4.63 (0.96;22.45)	0.057	7.12 (0.64;79.04)	0.110	59.31 (4.72;745.08)	**0**.**002**
Perineural growth	Yes vs. no	0.53 (0.16;1.80)	0.307	–	0.995	0.75 (0.25;2.24)	0.601
LVI	Yes vs. no	0.28 (0.04;2.08)	0.212	–	0.996	0.68 (0.16;2.94)	0.601
Surgical margins *(Ref = negative)*	Close	1.65 (0.55;4.92)	0.373	1.32 (0.15;11.95)	0.805	2.06 (0.60;7.07)	0.253
	Positive	1.83 (0.33;10.14)	0.489	2.80 (0.17;45.18)	0.469	3.49 (0.57;21.32)	0.176
DOI	+1	1.03 (0.95;1.13)	0.461	1.02 (0.86;1.22)	0.812	1.04 (0.96;1.13)	0.370

Significant with *p*-value less than 0.05 are indicated in bold.

## 4 Discussion

Despite the advancement of cancer therapy, the survival rate for OCSCC has not significantly changed over the past 20 years (10). Tumor behavior in patients is highly variable and depends on several host and primary tumor factors. Understanding these factors is important to estimate the prognosis of a patient and where possible intensify the initial treatment.

The 5-year survival rate for OCSCC across all stages ranges from approximately 60%–65% (1, 10, 17). Zhang et al. reported that in a retrospective cohort study of 343 patients with early-stage OCSCC who underwent primary surgery, the 5-year OS was 61.9%, while the 5-year DSS was 78.3% (18). In our study, the 5-year OS and DSS were 64.9% and 87.3%, respectively, which compare favorably to these results.

Of the 60 oral cancer patients included in this study, 39 (65%) were male and 21 (35%) female. This distribution is comparable to the male oral cancer prevalence in the United States, which is reported at 60.2% (19). In our study, male patients exhibited a significantly lower OS and a significantly shorter DFS compared to female patients. However, the literature presents conflicting findings, with some studies reporting no significant difference in survival between the sexes, while others do suggest a worse prognosis for male individuals with OCSCC compared to females, like we found in our cohort (20, 21).

Presentation with a second primary malignancy significantly worsened the prognosis of patients in our study (*p* = 0.029, HR = 0.35) (Figure 2). While some studies suggest no significant survival difference between primary and SPTs, Alvarez et al. reported a notably lower 5-year survival for patients with SPTs in the head and neck region (23% vs. 53% in control group) (22–24). In our study the 5-year OS in primary tumors was 73.4%, compared to 40% in SPTs. Several factors explaining this finding can be put forward. Second primary cancers develop independently in individuals previously diagnosed with and treated for cancer; a phenomenon explained by the concept of field cancerization. This theory, first introduced by Slaughter et al. in 1953, proposes that multiple malignancies can arise within a specific anatomical region characterized by tumor-associated genetic changes due to shared environmental exposures, such as tobacco use and prior radiation therapy (25).

While successful loco-regional control of oral cancer has improved patient outcomes, it has also contributed to the increasing incidence of SPTs. The relative risk of developing multiple primary cancers is higher in individuals that are younger when they develop the first primary, those who continue smoking and alcohol consumption after therapy for that primary, and patients treated with radiotherapy as part of their initial treatment. We found that active smoking status had a significant negative effect on OS (*p* = 0.0362), which may be related to an increased risk of developing a SPT (26). Multiple studies have demonstrated that smokers diagnosed with primary OCSCC have a higher risk of developing a second primary cancer (27).

The reported annual risk of developing a metachronous tumor ranges from 3% to 7%, with cumulative 5-year rates between 15% and 25%. In our study, we observed a slightly lower cumulative incidence, with 8 (13%) patients developing a second or third primary malignancy over a 12-year period (23). A study conducted in Southern England estimated that, within 20 years of an initial head and neck cancer diagnosis, approximately 30% of male patients and 20% of female patients will develop a SPT (26).

These malignancies are challenging to treat, complicated by the limitations imposed by the previous cancer treatment, making it challenging to follow conventional treatment guidelines, and further underscoring the importance of improving strategies for prevention and management. Current diagnostic methods have limitations, including the inability to diagnose in the early stages, which could be overcome by potential molecular techniques that use the expression of genetic variants *p53*, *p21*, *p73,* and glutathione S-transferase polymorphisms. Additionally, and not surprisingly, quitting smoking and alcohol may lower the risk of developing SPTs (27).

The TNM stage is widely recognized as a primary prognostic factor for OCSCC (19, 28, 29). In our study, the TNM staging system also proved to be a significant prognostic factor for DSS (*p* = 0.043, HR = 1.74). ENE, which is incorporated into the overall TNM staging, demonstrated a significant prognostic effect on both OS and DFS. Compared to the absence of ENE, gross ENE was significantly associated with worse OS (*p* = 0.048) and showed a borderline association with worse DFS (*p* = 0.050). Minimal ENE, while not significantly associated with OS or DSS, was strongly linked to worse DFS (*p* = 0.002). However, caution is warranted when interpreting these findings due to the wide confidence intervals. Additionally, the involvement of multiple (more than one) lymph nodes was significantly associated with worse OS. This finding is consistent with the results of a meta-analysis by Tsai et al., which demonstrated that lymph node burden (LNB), when considered as a continuous variable, was significantly correlated with poorer OS (30).

The study limitations include the retrospective study design, which inherently introduces potential selection and information biases. Additionally, the relatively small sample size limited the statistical power of our analyses and precluded a meaningful multivariate analysis. This underlines the necessity for future studies with larger cohorts that allow for adequately powered multivariate analyses. Such studies would be essential to control for potential confounding factors and to validate the trends observed in our exploratory analysis.

## 5 Conclusion

In conclusion, this study is innovative in identifying the presentation of OCSCC as of a second primary malignancy as an individual negative prognostic factor for OS, providing valuable insights for clinical decision-making and patient counseling. Furthermore, the univariate analysis identified male sex, TNM stage, gross ENE, LNB more than one, and active smoking status as significant indicators of poor outcomes. Larger studies with multivariate analyses are essential to improve treatment and prevention strategies for OCSCC (31).

## Data Availability

The raw data supporting the conclusions of this article will be made available by the authors, without undue reservation.
